# Trimodal therapy in T2‐4aN0M0 bladder cancer––How to select the best candidate?

**DOI:** 10.1002/cam4.3478

**Published:** 2020-09-22

**Authors:** Ofer N. Gofrit, Amichay Meirovitz, Stephen Frank, Igal Rabinovich, Hemda Luwisch, Vladimir Yutkin, Tzahi Neuman, Guy Hidas, Mordechai Duvdevani, Marc Wygoda

**Affiliations:** ^1^ Department of Urology Hadassah Hebrew University Medical Center Jerusalem Israel; ^2^ Department of Oncology Hadassah Hebrew University Medical Center Jerusalem Israel; ^3^ Department of Pathology Hadassah Hebrew University Medical Center Jerusalem Israel

**Keywords:** muscle‐invasive bladder cancer, patient selection, trimodal therapy, tumor diameter

## Abstract

The reported results of trimodal treatment (TMT) in muscle‐invasive bladder cancer vary widely. We attempted to characterize the profile of ideal candidates for this approach. Between 2000 and 2019, 105 patients (median age 78 years) with T2‐4aN0M0 bladder cancer were treated with TMT and analyzed retrospectively. Mean radiotherapy dose was 62 Gy (SD 8.4). Ten pretreatment prognostic parameters were evaluated including tumor diameter on pre‐TURBT CT. Multivariate analyses was performed and combination of parameters was studied. After a median follow‐up of 29 months, 53 patients (50.5%) developed recurrence and 70 patients (67.7%) died. Death was disease‐specific in 46 patients (65.7%). Tumor diameter was the most significant prognostic parameter with *p* < 0.0001 for overall, disease‐specific and recurrence‐free survivals. For every 1 cm increase in tumor diameter, the risk of disease‐specific mortality increased by 1.57. Age, cisplatin eligibility and the Charlson Comorbidity Index were significant predictors of overall survival but not of disease‐specific or recurrence‐free survival. Patients who were cisplatin‐eligible with a tumor diameter ≤3 cm had a 5‐year disease‐specific survival rate of 79.2% as opposed to 33.9% in patients without one of these features (*p* < 0.001). When tumor diameter exceeded 5 cm (irrelevant of all other parameters), 5‐year disease‐specific survival rate was only 28.2%. Patient profiles can accurately predict response to TMT. In cisplatin‐eligible patients with a tumor diameter ≤3 cm, TMT provides an excellent disease‐specific survival rate. In patients with a tumor diameter >5 cm TMT renders unacceptably poor treatment outcomes.

## Graphical abstract

Bladder tumor diameter on pretreatment CT is the single best predictor of tumor volume and of response to trimodal therapy. In cisplatin‐eligible patients with a tumor diameter ≤3 cm, trimodal treatment provides 5‐year disease‐specific survival of 80%

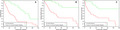

## 1 INTRODUCTION

Radical cystectomy (RC) with lymph node dissection preceded by cisplatin‐based neoadjuvant chemotherapy is currently the recommended treatment plan in patients with muscle‐invasive non‐metastatic bladder cancer.^1^ Bladder preservation with trimodal therapy (TMT), including maximal transurethral resection followed by chemo‐radiation, is an alternative treatment option but is not recommended as the standard therapy (level of evidence 2b).^1^, ^2^ The patient population referred for TMT is mixed and includes both fragile patients who are too ill to safely undergo RC and patients who are fit for surgery but are unwilling to accept its associated morbidity.

Trimodal therapy outcomes vary widely and are inclusive of very favorable results such as those reported from the Massachusetts General Hospital program showing 5‐year overall and disease‐specific survival rates of 57% and 66%, respectively.^3^ Similar results were reported from a pooled analysis of 6 RTOG trials with 5‐year overall and disease‐specific survival rates of 57% and 71%, respectively.^4^ Substantially lower rates are observed when larger databases from multiple centers are analyzed. In the “SEER” database the 5‐year overall survival and disease‐specific survival rates were 20% and 40%, respectively (40% and 60% after RC in that analysis).^5^ In the “Bladder Cancer Data Base Sweden” the rates were 23% and 49% (49% and 61% after RC) and in the National Cancer Database the 5‐year overall survival rate after TMT was 29.9% (48.3% after RC).^6^, ^7^ Selection bias explains at least some of the differences. For example, the median ages in the Massachusetts General Hospital program and in the combined RTOG studies were 66 and 67 years, respectively,^3^, ^4^ whereas the mean ages were 76 and 71 years in the SEER and Sweden databases, respectively. ^5^, ^6^

Candidates for TMT can be classified as either “medically operable” or “medically inoperable.” The literature suggests that positive outcomes for TMT are expected in “medically operable” patients with clinical T2, no hydronephrosis and complete TURBT.^8^, ^9^ The impact of CIS is less clear as it is considered in some manuscripts as a risk factor but not in others.^8^, ^9^ In study, using an “real‐life” population, we attempted to refine these prognostic criteria and to draw the profiles of optimal and suboptimal candidates for TMT.

## 2 METHODS

### 2.1 Patient cohort

Information was obtained retrospectively from the Hospital archives of patients with T2‐4aN0M0 bladder cancer treated between 2000 and 2019. The study was approved by the Institutional Review Board Committee.

Diagnosis of muscle‐invasive bladder cancer was made using TURBT specimens according to the AJCC TNM staging system, by a dedicated uropathologist. This diagnostic process initiated metastatic workup which included computed tomography of the chest, abdomen and pelvis and assessment of operative risk. Comorbidity was graded according to the Charlson Comorbidity Index.^10^ Patients with no evidence of metastases and a reasonable risk for general anesthesia were offered RC. TMT was considered in patients who were unfit for surgery due to comorbidity or unwilling to accept its associated morbidity. The potential prognostic parameters studied are presented in Table 1. All patients had urothelial carcinoma but 19 patients (18%) had metaplasia of portions of the tumor to one of the variants. Sarcomatoid features were found in only two patients and this parameter was not further analyzed. "Tumor diameter" was defined as the largest diameter of thickened bladder wall in pre‐TURBT CT. “Hydronephrosis” was defined as ballooning of the renal pelvis accompanied by dilatation of the ureter to the level of the bladder on pre‐TURBT CT. Data regarding "maximal TURBT" was obtained from the operative notes.

**TABLE 1:** Baseline characteristics of patients who underwent trimodal therapy for muscle‐invasive bladder cancer

Parameter	Value
Mean age in years (SD)	75.4 (10.6)
Median age in years (IQR)	78 (69‐82)
Male, gender (%)	76 (71.7%)
Mean Charlson Comorbidity Index (SD)	1.89 (1.15)
Primary/secondary tumors^a^	76/29 (71.7%/28.3%)
Hydronephrosis	24 (22.8%)
Mean maximal diameter on CT (SD)	3.8 cm (1.9)
Variant histology	19 (18%)
Sarcomatoid features	2 (1.9%)
Maximal transurethral resection	86 (81.9%)
Cisplatin as radiosensitizer	64 (61%)
Mean radiotherapy dose in Gy (SD)	62 (8.4)
Median follow‐up in months (IQR)	29 (15‐65)

^a^ ‘Primary tumors’ are first time tumors. Secondary tumors are stage a or stage 1 tumors that have progressed to muscle invasion during follow‐up.

### 2.2 Trimodal treatment

Chemo‐radiation was administered with curative intent following an attempt to maximally resect the tumor transurethrally. Cisplatin, at a dose of 40 mg/m^2^ weekly, was the preferred chemotherapeutic agent. In patients with compromised renal function, cisplatin was substituted with weekly carboplatin at AUC 2.

Similar radiotherapy technique was used along the years of the study with minor variations. It included IMRT modified according to various co‐factors (age, comorbidities, and tumor extent). In all cases, the entire bladder was included in the first part of the treatment. Doses ranged between 44 and 46 Gy in 1.8‐2 Gy fractions to the bladder/pelvic nodes followed by a boost of 16‐20 Gy (in 2 Gy fractions) bringing the total dose to 60‐66 Gy (mean 62 Gy, SD 8.4). Treatment planning was CT‐based after complete voiding of the bladder. Daily bladder image‐guidance was not done along most years of this study. All patients underwent at least a weekly portal film verification: in more recent years daily image matching was performed either through kV‐kV images or through Cone Beam CT.

Patient follow‐ups included evaluation of kidney function, cystoscopy and CT of the chest, abdomen and pelvis at 3 and 6 months post‐therapy completion, followed by 6 month intervals for 3 years, and then, at the clinician's discretion, with liberal use of suspicious lesion biopsies. Toxicity of therapy was graded according to the Common Terminology Criteria for Adverse Events v 4.0 (CTCAE) for chemotherapy and the RTOG toxicity grading systems for chemo‐radiation.^11^, ^12^ The highest grade is reported for each patient.

### 2.3 Statistical analyses

Statistical analysis was done using IBM SPSS STATISTICS, version 25.0 software. Descriptive statistics are presented as the mean with standard deviation (SD), or as the median with the interquartile range (IQR) based on the scale of the variable. Survival was calculated from the first treatment date. Disease recurrence was defined as either a recurrent muscle‐invasive tumor (or non‐muscle‐invasive tumor that cannot be controlled transurethrally), a new mass in the pelvis, lymph node enlargement or metastases in distant organs. Disease‐specific death was defined as death attributable to urothelial cell carcinoma (UCC) either according to a death certificate or being the main cause of death as per the hospital summary note, or as death due to UCC metastasis being unresponsive to treatment. Overall, disease‐specific and disease‐free survival rates were calculated using the Kaplan‐Meier method. The associations between all variables and survival rates were assessed using Cox regression models, where all variables with *p* < 0.2 were included in the multivariate Cox regression model. A *p*‐value <0.05 was considered statistically significant. All reported *p*‐values are two‐tailed and all the patients were included in the analysis.

## 3 RESULTS

A total of 105 patients were treated with TMT for T2‐4aN0M0 bladder cancer, their baseline characteristics are shown in Table 1. Their mean age was 75.4 (SD 10.6) and mean Charlson Comorbidity Index 1.89 (SD 1.15). Cisplatin as a radiosensitizer was given to 65 patients (61%). Other patients received various agents, most commonly carboplatin that was given to 24 patients (22.8%). TMT was tolerated relatively well with grade 3‐4 toxicity developing in only 26 patients (24.7%). Two patients developed end‐stage bladder and required cystectomy and urinary diversion. Viable tumor cells were not found in the pathological examination of their bladders.

After a median follow‐up of 29 months, 53 patients (50.5%) developed recurrence. In 20 patients (37.7%) recurrence was loco‐regional only, in 23 patients (21.9%) recurrence was systemic only and in 10 patients (9.5%) recurrence was both systemic and loco‐regional. Salvage cystectomy was done in 6 patients (5.7%). Reconstruction of the urinary system was accomplished with an ileal conduit in all of them. Pathology showed T3 disease in three patients, T1 in two and T0 in one. Therapy in cases of recurrence included chemotherapy or immunotherapy in 16 patients (20.9%) and supportive care in the rest.

**FIGURE 1:**
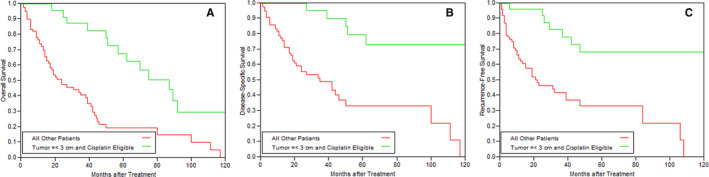
Kaplan‐Meier curves of (a) overall, (b) disease‐specific and (c) recurrence‐free survival rates of cisplatin‐eligible patients with tumors ≤3 cm and of patients without one of these features

During the follow‐up process 70 patients (68.6%) died. In 46 patients (65.7%), death was disease‐specific. Median overall survival was 3.2 years, with 2 and 5 year overall, disease‐specific and recurrence‐free survival rates of: 60.2% and 32.5%, 67.6% and 44.8%, and 60.4% and 43.4%, respectively. Overall, disease‐specific and recurrence‐free survival rates and their univariate and multivariate dependence on pretreatment parameters are presented in Tables 2 and 3. In multivariate analysis pretreatment tumor diameter was the most significant prognostic parameter (*p* < 0.0001 for overall, disease‐specific and disease‐free survival rates). Every increase in tumor diameter by 1 cm increased the hazard of overall mortality by 1.37, disease‐specific mortality by 1.57 and disease recurrence by 1.59. Age, cisplatin eligibility and Charlson Comorbidity Index were significant predictors of overall survival but not of disease‐specific or recurrence‐free survival rates. Table 4 presents various profiles of patients and their survival rates. Cisplatin‐eligible patients with a tumor diameter ≤3 cm and no hydronephrosis had a 5‐year disease‐specific survival of 83.6%. If hydronephrosis is omitted from this profile (Figure 1), the 5‐year disease‐specific survival remains remarkably high at 79.2% as opposed to 33.9% in patients without one of these features (*p* < 0.001). When tumor diameter exceeded 5 cm (irrelevant of all other parameters), 5‐year disease‐free survival was only 28.2%.

**TABLE 2:** Univariate analysis of the effect of various pretreatment parameters on overall, disease‐specific, and recurrence‐free survival rates after trimodal therapy for muscle‐invasive bladder cancer (CI, confidence interval; HR, hazard ratio)

Parameter	Overall survival		Disease‐specific survival		Recurrence‐free	
	*p*‐value	HR (95% CI)	*p*‐value	HR (95% CI)	*p*‐value	HR (95% CI)
Age	<0.0001	1.058 (1.03‐1.08)	0.016	1.038 (1.01‐1.07)	<0.1	1.022 (0.99‐1.05)
Gender	0.7	0.902 (0.53‐1.52)	0.772	1.104 (0.56‐2.17)	0.64	0.902 (0.53‐1.53)
Charlson Index	0.057	1.234 (0.99‐1.53)	0.657	1.065 (0.80‐1.40)	0.83	1.028 (0.79‐1.32)
Hydronephrosis	0.096	1.585 (0.92‐2.72)	0.008	2.418 (1.31‐4.47)	0.01	2.15 (1.19‐3.88)
Tumor diameter	<0.0001	1.37^a^ (1.20‐1.56)	<0.0001	1.57^a^ (1.34‐1.84)	<0.0001	1.59^a^ (1.37‐1.85)
Prim/Sec disease	0.562	1.163 (0.69‐1.93)	0.686	1.14 (0.60‐2.15)	0.332	1.37 (0.72‐2.6)
Maximal TURBT	0.16	0.65 (0.37‐1.18)	0.041	0.48 (0.25‐0.93)	0.01	0.442 (0.24‐0.82)
Variant histology	0.640	0.862 (0.46‐1.60)	0.993	1.00 (0.48‐2.1)	0.89	0.96 (0.462‐1.608)
Cisplatin eligible	0.002	0.468 (0.28‐0.76)	0.112	0.61 (0.33‐1.1)	0.002	0.468 (0.48‐0.89)

^a^ For every centimeter of tumor.

**TABLE 3:** Multivariate analysis of the effect of various pretreatment parameters on overall, disease‐specific, and recurrence‐free survival after trimodal therapy for muscle‐invasive bladder cancer (CI, confidence interval; HR, hazard ratio)

Parameter	Overall survival		Disease‐specific survival		Recurrence‐free	
	*p*‐value	HR (95% CI)	*p*‐value	HR (95% CI)	*p*‐value	HR (95% CI)
Age	0.001	1.05 (1.02‐1.08)	0.035	1.033 (1.00‐1.06)	0.18	1.02 (0.99‐1.05)
Hydronephrosis	0.11	1.63 (0.89‐2.98)	0.07, 1.86 (0.95‐3.63)	0.32	1.39 (0.72‐2.71)	
Tumor Diameter	<0.0001	1.41^a^ (1.24‐1.61)	<0.0001	1.49^a^ (1.23‐1.70)	<0.0001	1.51^a^ (1.29‐1.76)
Cisplatin eligible	0.03	0.55 (0.32 −1.62)	0.18	0.63 (0.32 −1.24)	0.60	1.60 (0.32 −1.14)
Charlson Index	0.01	1.35 (0.32 −0.94)	—	—	—	—
Maximal TURBT	—	—	0.165	0.59 (0.28 −1.24)	—	—

^a^ For every centimeter of tumor.

**TABLE 4:** Various pretreatment profiles of patients and their effects on overall, disease‐specific, and recurrence‐free survival rates after trimodal therapy for muscle‐invasive bladder cancer

Parameter	Number of Patients	Overall survival		Disease‐specific		Recurrence‐free	
		2‐year	5‐year	2‐year	5‐year	2‐year	5‐year
Tumor ≤3 cm	47	67.5%	51.3%	76.4%	49.9%	65.4%	43.6%
Tumor >3 cm	58	53.3%	22.6%	59.1%	42.5%	53.1%	45.9%
*p* value		0.039		0.123		0.36	
Tumor ≤5 cm	77	65.9%	37.6%	75.5%	55.4%	69.9%	51.2%
Tumor >5 cm	28	45.2%	20.6%	48.2%	28.2%	32.1%	24.1%
*p* value		0.03		0.0015		0.0006	
Hydronephrosis—No	81	59.5%	33.4%	67.2%	49.5%	58.9%	46.7%
Hydronephrosis—Yes	24	61.7%	29.4%	68.4%	39.5%	60.0%	32.4%
*p* value		0.56		0.47		0.39	
Cisplatin‐Eligible—yes	64	63.3%	39.6%	70.1%	48.2%	60.2%	39.8%
Cisplatin‐Eligible—No	41	52.8%	16.5%	63.6%	45.2%	56.9%	52.1%
*p* value		0.125		0.67		0.62	
Tumor ≤3 cm and Cisplatin‐Eligible	27	91.1%	62.2%	100%	79.2%	91.6%	67.5%
Tumor >3 cm or Cisplatin‐Ineligible	78	48.4%	19.9%	56.0%	33.9%	47.3%	34.1%
*p* value		<0.001		<0.001		0.002	
Tumor ≤5 cm and Cisplatin‐Eligible	27	77.6%	51.6%	80.3%	59.1%	70.7%	51.2%
Tumor >5 cm or Cisplatin‐Ineligible	78	48.4%	20.1%	58.0%	36.9%	50.1%	36.9%
*p* value		0.002		0.018		0.032	
Tumor ≤3 cm and Cisplatin‐Eligible and no Hydronephrosis	22	90.5%	70.6%	100%	83.6%	95%	73.9%
Tumor >3 cm or Cisplatin‐Ineligible or hydronephrosis	83	50.0%	19.6%	57.5%	67.7%	48.4%	33.3%
*p* value		<0.001		<0.001		<0.001	

## 4 DISCUSSION

Is RC or TMT the best therapeutic approaches for patients with muscle‐invasive bladder cancer. Direct comparison in a prospective randomized trial would be expected to provide the answer. The SPARE (Selective Bladder Preservation Against Radical Excision) trial attempted to do that, however, poor study accrual due to clinician and patient preference for treatment brought its premature closure.^13^ When addressing whether retrospective comparisons may provide reliable answers, it must be noted that these comparisons are always subject to selection bias that can only be partially corrected with statistical tools. For example, Cahn et al. studied the National Cancer Database and compared the outcome of 22,680 patients who underwent RC to 2540 patients treated with radiotherapy, where only 1489 also received chemotherapy (TMT).^7^ Five‐year overall survival was 48.3% after RC and 29.9% after TMT (HR 1.578, 95% CI 1.477‐1.686). However, patients referred to TMT were significantly older (76.6% of them were older than 71 years compared to only 49.7% in the RC group) and had a significantly higher Charlson score as compared to those who received RC. When multivariate matched propensity score was applied, this difference decreased (HR 1.46, 95% CI 1.235‐1.601). By comparing propensity matched cohorts from the same database, Zhong et al. showed that patients after TMT and RC had similar median overall survival rates (2.7 years vs. 3 years *p* = 0.2). ^14^ Nevertheless, matched propensity methods cannot adjust for unknown or difficult to measure confounders that affect patient and physician discretion when deciding which treatment to choose.

In the current study, median overall survival was 3.2 years, with 5‐year overall and disease‐specific survival rates of 32.5% and 44.8%, respectively. These results are similar to the outcomes reported in large contemporary studies.^5^, ^6^, ^14^ Ten potential prognostic pretreatment parameters were evaluated including the novel "tumor diameter," which proved to be the most influential. Every increase in tumor diameter by 1 cm increased the hazard of disease‐specific mortality by 1.57. Other known prognostic parameters such as the presence of hydronephrosis and maximal TURBT proved less importance as prognosticators. Gender, the presence of variant histology and primary vs. secondary tumors had no influence on prognosis.

Tumors with identical stage classifications can vary by a factor of more than 100 in volume. Tumor volume is one of the classical predictors of response to radiotherapy, with clinical and experimental data supporting this.^15^ Tumor volume is a strong predictor of the hypoxic fraction of the tumor, the number of clones that have to be sterilized and its doubling time, with multiple examples of its importance in other cancers. In stage IB cervix cancer, tumor diameter highly correlates with disease‐specific survival (*p* < 0.0001).^16^ In breast cancer, the risk of local recurrence after radiotherapy alone correlates with tumor diameter (*p* < 10^−6^).^17^ In head and neck cancer, tumor and node volumes are strong prognosticators of survival (*p* = 0.0003) and loco‐regional control (*p* = 0.0002).^18^ Finally, in melanomas, tumor volume correlates with complete response rate after radiotherapy (*p* < 0.001).^19^

Hydronephrosis and maximal TURBT are both surrogates of tumor volume, and therefore, influence the response of bladder cancer to radiotherapy. A tumor, however, at the dome of the bladder can be very extensive without obstructing a ureteral orifice and "maximal TURBT" is a subjective impression of the surgeon. Tumor diameter, on the contrary, is an objective factor that is easily measured, and it is not clear why such a simple and important parameter has been overlooked thus far. The largest tumor diameter on a single CT slice was used as an indicator of tumor volume. Calculation of tumor volume from multiple slices, however, as reported by Johnson et al. in head and neck cancer, may yield an even more accurate estimation of the volume but is less accessible to the clinician.^20^ An even better estimate of tumor's volume by volume calculation with MRI as was already reported in Glioblastomas.^21^

Combining several parameters into a “profile” of a patient was found to be highly predictive (Table 4; Figure 1). In cisplatin‐eligible patients with a tumor diameter ≤3 cm and no hydronephrosis, the 5‐year disease‐specific survival rate was 83.6%. Even when hydronephrosis was omitted from this profile, the 5‐year disease‐specific survival rate remained remarkably high (79.2%). These figures closely match the results of RC in patients with pathologically confirmed T2 stage cancer as reported by Shariat et al. despite the age difference between the study cohorts (mean age of 66.2 years in the study by Shariat et al. and 75.4 years in the current study).^22^ Thus, patients who are cisplatin‐eligible with tumors ≤3 cm may safely consider TMT as a treatment option without compromising prognosis. Conversely, when tumor diameter was larger than 5 cm the results of TMT were extremely poor with 5‐year disease‐specific survival rate of 28%. It must be remembered that large tumor diameter (>3 cm) is a poor prognostic factor also after RC.^23^, ^24^

### 4.1 Limitation

This study has several limitations inherent to retrospective analysis. Assigning cause of death in retrospect could have resulted in an ascertainment bias, the cohort was relatively small and it is possible that with a larger cohort, parameters that were found to be insignificant, such as hydronephrosis for disease‐specific survival (*p* = 0.07), would become significant. We used pre‐TURBT tumor's largest diameter on CT as a surrogate of tumor's volume. Assessment of residual pre‐radiotherapy tumor could possibly provide more prognostic information. The inclusion of molecular markers for predicting response to treatment may allow for more precise estimations in the future.^1^ It Is possible that twice weekly gemcitabine at 27 mg/m^2^, instead of carboplatin AUC 2 used here, could improve the outcome of cisplatin ineligible patients. A trial with low dose cisplatin or gemcitabine with or with pembrolizumab (MSD 992 trial) is recruiting patients and may impact on our approach to TMT.

### 4.2 Conclusions

This study showed that pre‐TURBT tumor diameter is the best preoperative predictor of response to TMT. Combining tumor diameter with cisplatin‐eligibility creates a powerful predictive profile. In this study, if a patient was cisplatin‐eligible and had a tumor ≤3 cm, TMT provided an 80% 5‐year disease‐specific survival rate, which is equivalent or even superior to that which is obtained with RC. Conversely, if tumor diameter was larger than 5 cm the prognosis after TMT was poor.

## CONFLICT OF INTEREST

None.

## Data Availability

All data are available for review (as an Excel file).
